# Who eats whom? Interactions between the non-native snail *Physa acuta*, local digeneans, and a commensal oligochaete

**DOI:** 10.1017/S0031182025101169

**Published:** 2026-01

**Authors:** Anna Stanicka, Jarosław Kobak, Zuzanna Kowaleska, Monika Lewalska, Wiktoria Pacek, Arkadiusz Grzeczka, Szymon Graczyk, Anna Cichy, Elżbieta Żbikowska

**Affiliations:** 1 Department of Invertebrate Zoology and Parasitology, Nicolaus Copernicus University in Toruń, Toruń, Poland; 2 Department of Preclinical and Basic Sciences, Nicolaus Copernicus University in Toruń, Toruń, Poland; 3 Department of Diagnostic and Clinical Sciences, Nicolaus Copernicus University in Toruń, Toruń, Poland

**Keywords:** free-living larval stages, non-host effects, non-native species, parasite suppression, parasite transmission, predator–parasite interaction

## Abstract

Parasite transmission can be disrupted when their free-living larval stages are consumed by non-host organisms. Yet, the contribution of benthic scrapers to this process remains insufficiently explored. Here, we experimentally assessed the ability of the North American pulmonate snail *Physa acuta* to reduce the abundance of free-living digenean larvae – cercariae of *Diplostomum* sp. and *Trichobilharzia* sp., and adolescariae of *Notocotylus* sp. – and evaluated how this effect is modulated by snail body size and colonisation by other organisms. Larval consumption by *P. acuta* occurred in all treatments and was highest for settled *Notocotylus* sp. adolescariae, particularly among larger individuals. The extent of larval reduction varied with infection by digenean metacercariae (xiphidiometacercariae), which either enhanced or inhibited feeding depending on parasite identity. It also varied with colonisation by *Chaetogaster limnaei limnaei*, whose presence increased the ingestion of planktonic cercariae, likely due to the combined feeding activity of the snail and its commensal oligochaete. Most snails harboured metacercariae, indicating that *P. acuta* frequently functions as a second intermediate host in its non-native range. Our findings highlight the dual ecological role of *P. acuta* – both as a consumer of free-living parasite stages and as a competent host. This trophic interaction may disrupt parasite transmission while providing nutritional benefits that support the ecological success and spread of this non-native species. Conversely, by serving as a host, *P. acuta* may facilitate the persistence and dissemination of parasitic taxa in invaded ecosystems.

## Graphical abstract

**Figure:**
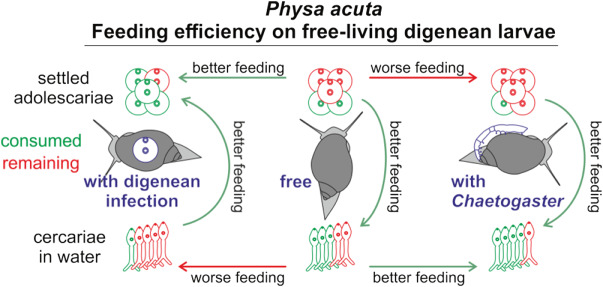

## Introduction

Among multiple ecological consequences of biological invasions, the emergence of novel interspecific interactions and the alteration of existing ones are considered particularly important in the context of global change. These processes can destabilise ecological balances in local communities, ultimately leading to declines in native species (Jeschke et al. 2014; Wauters et al. 2023). Parasites often play a role in such interactions: they may expand their range with introduced hosts, infect new local host species, or broaden their host spectrum due to the arrival of novel, suitable hosts (Dunn et al. 2012; Schatz and Park 2023; Díaz-Morales et al. 2025). In addition to these direct parasitic interactions, indirect trophic relationships between non-native species and free-living parasite stages also emerge as ecologically relevant yet understudied mechanisms (Stanicka et al. 2023; Díaz-Morales et al. 2025). These phenomena are less predictable and harder to observe. They include dilution effects, whereby parasite transmission is disrupted by the presence of non-host organisms, the latter often being non-native members of the local community (Stanicka et al. 2025). One important mechanism behind this effect involves the removal of free-living larval parasite stages (e.g. digenean cercariae) from the environment through their consumption (Koprivnikar et al. 2023; Rosen et al. 2025). The ability to feed on these larvae appears to be highly species-specific (Hopper et al. 2008; Welsh et al. 2019), depending on both parasite dispersal strategies and foraging behaviours of potential consumers (Selbach et al. 2019). Although trematode larvae constitute a potential energy-rich food source (McKee et al. 2020), their role in supporting the ecological success of their non-native consumers has received limited scientific attention.

The freshwater pulmonate snail *Physa acuta* (Draparnaud, 1805) (Physidae), native to North America and now distributed globally, appears to be a suitable candidate for exploring trophic interactions between non-host consumers and free-living parasite stages. Depending on the region, it is considered a non-native or even invasive species (Cieplok and Spyra 2020). Its success is attributed to broad environmental tolerance, resistance to pollution, an omnivorous diet, and high reproductive output (Piechocki and Wawrzyniak-Wydrowska 2016). In some European habitats, e.g. in Poland, population densities can reach up to 4000 individuals per square metre (Spyra et al. 2019). Due to its widespread occurrence and ecological plasticity, *P. acuta* has become a model organism in ecological and parasitological research. Given its ecological ubiquity and feeding strategy, *P. acuta* is not only a potential host for local parasites and a vector for alien ones, but may also affect parasite populations through other mechanisms, such as predation on their free-living stages (Stanicka et al. 2021a).

Gastropods are integral to the life cycles of digenean trematodes (Digenea), which usually rely on molluscs as their first intermediate hosts (Faltýnková et al. 2008). Although *P. acuta* is rarely exploited as a first intermediate host by digenean trematodes in its non-native range, it has been recorded as a second intermediate host (Pantoja et al. 2021). Its regular co-occurrence with free-living trematode larvae in invaded habitats suggests it may influence parasite transmission through other mechanisms. One plausible mechanism is the direct consumption of larvae while grazing periphyton from submerged surfaces (Selbach et al. 2019). Free-living digenean larvae are known to be consumed by active predators and filter feeders (Koprivnikar et al. 2023), yet little is known about their role in the diet of scrapers, such as *P. acuta* (Selbach et al. 2019; Stanicka et al. 2021a). Energy reserves of free-living digenean stages are limited, resulting in a short lifespan (Marszewska et al. 2016). Before dying, cercariae reduce their motility, shed their tails, and gradually sink to the bottom, eventually undergoing rapid body disintegration (Braun et al. 2018). It is therefore possible that *P. acuta* primarily feeds on such exhausted and dying cercariae near the substrate. As a scraper, this snail may find such immobile prey easier to access and energetically less costly to consume. Importantly, the consumption of larvae that have already lost their infective capacity does not contribute to the dilution effect. Instead, it would provide a unilateral advantage to the consumer, offering a readily available and nutritionally valuable source of proteins and energy. On the other hand, it cannot be a priori excluded that snails may be capable of feeding on live, vigorous cercariae. Moreover, they may feed on sedentary larval types, such as adolescariae. This would contribute to the dilution of affected digenean species in the environment. Therefore, it is important to determine whether *P. acuta* is also capable of feeding on live, infective digenean larvae.

To better understand how interactions with non-host organisms might affect digenean transmission, it is important to briefly outline the biology of their free-living larval stages. Digeneans have complex life cycles involving several hosts, and their free-living stages, such as miracidia and cercariae, are exposed to various ecological pressures, including predation and competition (Johnson et al. 2010). Miracidia, the first larval stage, actively or passively infect the first intermediate host, typically a snail. Cercariae, in turn, are released into the environment in large numbers and seek the next host. In some species, a third free-living larval stage – adolescaria – develops from a cercaria. This encysted metacercarial form attaches to submerged surfaces in the environment, bypassing the second intermediate host (Simon-Vicente et al. 1985; Galaktionov and Dobrovolskij 2013; Gonchar et al. 2019). Notably, adolescariae are also present in the life cycles of species of medical and veterinary relevance, such as *Fasciola hepatica* Linnaeus, 1758 and *Fasciolopsis buski* (Lankester, 1857) (Fasciolidae) (Mas-Coma et al. 2015).

Apart from digeneans themselves, other invertebrates also interact with freshwater snails, potentially affecting their relations with parasites. A well-known case is the oligochaete *Chaetogaster limnaei limnaei* Baer, 1827. This species, considered commensal or mutualist, is found worldwide and typically resides in the mantle cavity of its snail host (including *P. acuta*), feeding on mucus and microorganisms (Stoll et al. 2013; Okeke and Ubachukwu 2017; Bashê 2023). Importantly, *C. limnaei limnaei* can also prey on free-swimming digenean larvae, reducing infection success in the host snail (Hobart et al. 2021). Although some negative effects of *C. limnaei limnaei* on the snail have been observed in experimental settings, such situations seem to be rare (Stoll et al. 2013).

This study aimed to determine whether *P. acuta* can reduce the number of free-living larvae of digenean trematodes. We hypothesised that larval reduction would be detectable and depend on larva type and consumer size. Specifically, larger snails were expected to consume more than smaller individuals, and a greater reduction was anticipated for substrate-attached adolescariae compared to actively swimming cercariae. We also assumed that colonisation of *P. acuta* by other organisms could influence consumption of larvae: trematode metacercariae were expected to impair their host snail’s consumption efficiency, while the presence of the commensal *C. limnaei limnaei* might enhance larval removal.

## Material and methods

### Experimental prey

Free-living larvae of digenean trematodes were used as prey. These were cercariae of *Diplostomum* sp. (Diplostomidae) and *Trichobilharzia* sp. (Schistosomatidae), as well as adolescariae of *Notocotylus* sp. (Notocotylidae).

Among the digenean representatives used, the genus *Diplostomum* usually shows the highest prevalence in mollusc hosts in European waters (Faltýnková and Haas 2006; Soldánová et al. 2011; Stanicka et al. 2022). It is characterised by a three-host life cycle and uses various fish species as second intermediate hosts, which makes it important from the ecological, economic, and veterinary point of view (Antychowicz et al. 2016). Diplostomiasis poses a significant risk to fish farming, severely affecting the visual organ’s function in fish (Karvonen and Seppälä 2008).

The genus *Trichobilharzia*, although widespread and commonly recorded in snail hosts, usually reaches a relatively low prevalence (Soldánová et al. 2013; Stanicka et al. 2022). In their two-host life cycle, they use aquatic birds as definitive hosts. They are of medical significance due to cercariae causing a persistently itchy rash (swimmers’ itch) in humans, who serve as dead-end hosts (Marszewska et al. 2016).

*Notocotylus* spp. are widely distributed in marine, brackish, and fresh waters (Cribb 1991; Flores et al. 2023). They have a two-host life cycle, with a specifically short cercarial stage (Morley et al. 2002) and a free-living adolescaria infecting the definitive host, an aquatic bird (including domestic fowl) or mammal, by ingestion. Adults can induce intestinal pathologies in their hosts, leading even to their death (Graczyk and Shiff 1993).

Cercariae were obtained from naturally infected *Lymnaea stagnalis* collected from Lake Licheńskie (Poland, 52°19′26.6″N, 18°20′55.9″E). Adolescariae originated from naturally infected *Planorbarius corneus* (Linnaeus, 1758) (Gastropoda: Planorbidae) obtained from Lake Iławskie (Poland, 53°35′42.5″N, 19°37′07.2″E). The snails were collected in the second half of summer.

We placed infected molluscs in beakers with a small amount of dechlorinated tap water under an artificial light source (desk lamp). Cercariae of *Diplostomum* sp. and *Trichobilharzia* sp. that emerged into the water column were pipetted under a stereomicroscope (Science ETD-101, Bresser, Germany). Adolescariae were collected from the bottom and sides of the beakers, to which *Notocotylus* sp. cercariae were released. The larvae were used in the experiments no later than 3 hours after their release. Preliminary tests conducted during the development of the experimental protocol, along with literature data on larval survival (Karvonen et al. 2003; Al-Jubury et al. 2020), indicate that this timeframe ensured their viability throughout the experiment.

### *Sampling and acclimation of* Physa acuta

We collected *P. acuta* from Lake Licheńskie (Poland, 52°19′26.6″N, 18°20′55.9″E) in the second half of summer. First, the snails were kept individually in beakers with dechlorinated tap water for two hours. This incubation aimed to check for the release of cercariae, thus excluding patent digenean infections in the experimental snails. Next, *P. acuta* individuals were weighed using an electronic laboratory balance (AS 110/X, RADWAG, Poland) and divided into two size groups: 1) small – 0.05 g (SE ± 0.002 g), and 2) large – 0.12 g (SE ± 0.003 g). Finally, they were kept individually in beakers for 48 hours without access to food to acclimate them to the experimental thermal conditions (20 °C).

### Experimental design and procedure

**Figure 1.**
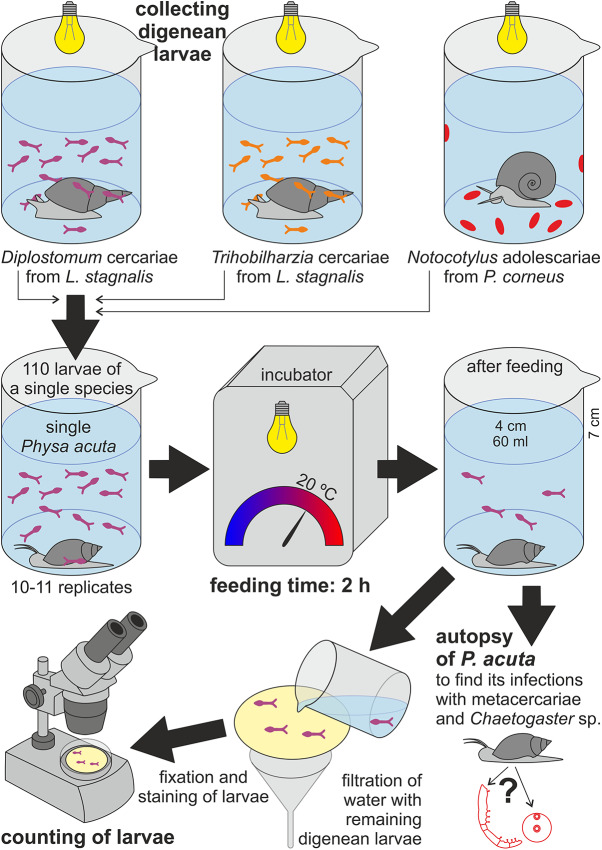
Schematic diagram of the experimental setup.

We placed individual *P. acuta* in experimental beakers (height × diameter: 70 mm × 40 mm) with a specified number of digenean larvae of a given species (110 individuals in 60 mL of water). The beakers were placed in a MIR 253 incubator (Sanyo, Japan) for 2 hours at 20 °C with artificial lighting (Figure 1). A control variant was included using larvae of each parasite species incubated in the absence of *P. acuta*. Each variant (experimental/control) was replicated 10-11 times. In total, 65 snails and 3520 digenean larvae were used in the experiment.

After incubation, *P. acuta* were immediately removed from the beakers and examined under a stereomicroscope to check for the presence of any larvae on their body surface (Stanicka et al. 2021a). The contents of each beaker were then filtered using a filtration set (Filter holder with receiver PSF, Thermo Scientific, USA) with a hand vacuum pump (Mityvac MV8010 Selectline Hand Vacuum Pump, SKF, Sweden). Subsequently, the inner walls of each beaker were rinsed twice with clean water to ensure that no digenean larvae remained attached, and the rinsing suspension was also filtered. Digenean larvae were retained on the surface of membrane filters with a diameter of 47 mm and a pore size of 12.0 μm (Whatman Cyclopore Polycarbonate Track-Etched Membrane (PCTE), Cytvia, Germany) (Figure 1). Before counting, the larvae were preserved in 70 % ethanol and stained (Nile Blue A, Sigma, USA) (Born-Torrijos et al. 2021). Finally, *P. acuta* were necropsied to look for parasites and commensals (Stanicka et al. 2021b).

### Data analysis

We analysed digenean larvae consumption by snails (a fraction of consumed individuals) using a Generalised Linear Mixed Model (GLMM) with binomial distribution and logit link function, including (1) digenean larva taxon present in water (*Diplostomum* sp., *Trichobilharzia* sp., *Notocotylus* sp.) and (2) snail size group (small/large) as categorical factors. Moreover, abundances of the most common parasites and commensals detected in experimental snails: (3) xiphidiometacercariae (Digenea: Plagiorchiida) and (4) *C. limnaei* Baer, 1827 (Annelida: Oligochaeta) were included as continuous covariates. Echinostome and tetracotyle metacercariae were also detected in experimental snails, but their presence was not considered in the analyses due to their low abundance. Finally, (5) snail individual was included in the model as a random factor. The loss of larvae in control trials without snails was negligible (see Results), therefore, possible losses were not taken into account in further analyses.

Initially, we included all main fixed effects and their interactions in the model. Then, we conducted a model simplification by removing non-significant highest-order interactions. As needed, for significant main effects of categorical factors and their interactions, we conducted pairwise contrasts as a post-hoc procedure. For significant interactions between categorical factors and covariates, we first checked the significance of regression slopes for each level of the categorical factor and then compared significant slopes with each other.

The analyses were run using SPSS 29.0 statistical software (IBM Corporation).

## Results

### *Consumption of vigorous free-living digenean larvae by* Physa acuta

In control vessels, digenean larva reduction ranged between 0.5% (*Notocotylus* sp. adolescariae) to 2% (cercariae of both species), indicating that their mortality due to other reasons than snail predation was negligible.

The reduction in the number of digenean larvae relative to their initial abundance in the vessel (consumption) was observed in all experimental variants. Further evidence supporting the potential feeding of *P. acuta* on digenean larvae is the detection of numerous *Notocotylus* sp. adolescariae in the digestive system of experimental snails (Fig. SM1).

**Figure 2.**
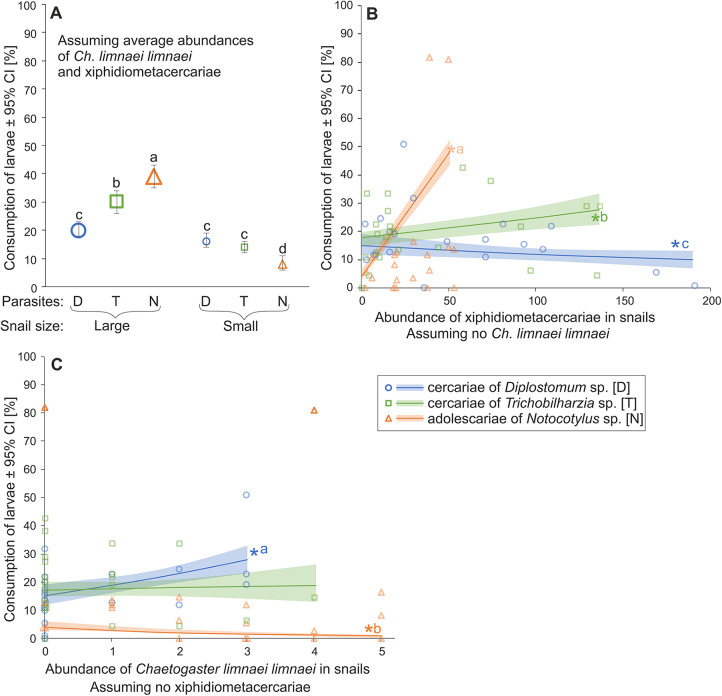
Consumption of digenean larvae by small and large *Physa acuta*, depending on larva species (A), infection of snails with xiphidiometacercariae (B) and presence of the oligochaete commensal *Chaetogaster limnaei limnaei* in snail mantle cavity (C). Different letters in panel A indicate significant differences between experimental treatments. Asterisks in panels B and C indicate a significant slope, while different letters show significant differences between the slopes.

Consumption of digenean larvae by snails (Figure 2A) depended on two-way interactions of digenean larva taxon with snail size group and abundances of xiphidiometacercariae and *C. limnaei limnaei* in snails (Table 1). A digenean larva taxon*snail size group interaction (Table S1A) resulted from large snails consuming more *Notocotylus* sp. adolescariae compared to the other digenean larvae. In contrast, small snails consumed them less efficiently than cercariae. Moreover, large snails generally consumed more digenean larvae (all species) than small individuals. Also, large snails consumed more cercariae of *Trichobilharzia* sp. than those of *Diplostomum* sp.

**Table 1.** Generalised Linear Mixed Model to test consumption of digenean larvae by snails depending on digenean taxon, snail size group (small/large) and abundance of xiphidiometacercariae and *Chaetogaster limnaei limnaei* present in the snails. The model also included a snail individual as a random factor (not shown). The highest order non-significant interactions were dropped in a model simplification procedure

Effect	Wald *χ*^2^	df	*P*
Digenean larva taxon (D)	48.80	2	<0.001
Snail size group (S)	7.35	1	0.007
Xiphidiometacercariae (X)^cov^	115.24	1	<0.001
*Chaetogaster limnaei limnaei* (Ch)^cov^	1.04	1	0.307
D × S	64.44	2	<0.001
D × X	131.18	2	<0.001
D × Ch	74.30	2	<0.001

cov – continuous covariate.

Consumption of digenean larvae by snails depended on their infection level and type (Table 1). Infection by xiphidiometacercariae (Figure 2B) increased consumption of *Notocotylus* sp. and *Trichobilharzia* sp., but reduced that of *Diplostomum* sp. (Table SM1B). On the other hand, infection by *C. limnaei limnaei* (Figure 2C) increased consumption of *Diplostomum* sp., decreased consumption of *Notocotylus* sp. and did not affect that of *Trichobilharzia* sp. (Table SM1C).

### Parasites and symbionts of *Physa acuta*

Only one individual of *P. acuta* did not serve as a second intermediate host for digenean metacercariae in our experiment. The total number of metacercariae was 2462, of which nearly 99% were xiphidiometacercariae (prevalence: 98%, mean infection intensity: 40 metacercariae per snail) (Fig. SM2). The remaining 1% comprised echinostome and tetracotyle metacercariae, with a prevalence of 18 % and 7%, and a mean infection intensity of two and one individuals per snail, respectively (Table 2). Additionally, over half of the examined snails (52%) were colonised by *C. limnaei limnaei*, with a total number of 79 individuals. The mean colonisation intensity of *P. acuta* by these oligochaetes was two individuals per snail (Table 2).

**Table 2.** Prevalence and mean infection intensity of metacercariae and *Chaetogaster limnaei limnaei* in *Physa acuta*

Digeneans/Oligochaetes^a^	*Physa acuta* size group	Prevalence [%]	Total prevalence [%]	Mean infection intensity (± SE)	Total mean infection intensity (± SE)	Min-max number
X	Large	100	98	39 (±7.08)	40 (±5.39)	2−169
	Small	97		42 (±8.50)		0−191
E	Large	23	18	2 (±0.34)	2 (±0.46)	0−3
	Small	13		2 (±1.19)		0−6
T	Large	7	7	1 (±0)	1 (±0.25)	0−1
	Small	7		1 (±0.5)		0−2
C	Large	50	52	2 (±0.41)	2 (±0.23)	1−5
	Small	56		2 (±0.25)		1−4

a X – xiphidiometacercariae, E – echinostome metacercariae, T – tetracotyle metacercariae, C – *Chaetogaster limnaei limnaei.*

## Discussion

Our tests indicate that *P. acuta* can feed on live, free-living digenean larvae, including those actively swimming in the water column (cercariae). However, the efficiency of feeding depends on the snail’s size, the larval species, and the presence of other parasites – for example, larger individuals consume *Notocotylus* sp. adolescariae more effectively, and the level of metacercarial infection may increase or decrease the consumption of specific larval taxa. Additionally, our study suggests a potential role of *C. limnaei limnaei*, a commensal inhabiting *P. acuta*, as an additional diluter of free-swimming cercariae. These findings highlight that the presence of the non-native *P. acuta* may disrupt parasite transmission, potentially benefiting host organisms, and, in the case of parasites of medical or veterinary importance, also human welfare. In turn, an important next research question emerges as a result of our findings: can the consumption of digenean cercariae by *P. acuta* contribute to an increase in its condition and reproductive success, and, consequently, facilitate its establishment and further spread as a non-native species? Given the widespread and continuous presence of digenean cercariae in aquatic environments, this potential advantage may play a significant role in invasion dynamics and warrants further investigation. Finally, our findings confirmed that *P. acuta* serves as an intermediate host for digenean metacercariae in non-native regions, broadening the host range and potentially facilitating the spread of parasitic species.

### Consumption of various types of digenean prey by snails

Differences in the consumption of various types of larvae can be explained by linking their behaviour and vertical distribution in the water column to the feeding strategy of *P. acuta*. Food located on the substratum is more accessible to scrapers than material suspended in the water column (Piechocki 1979). In our study, food associated with the bottom included adolescariae of *Notocotylus* sp. (Galaktionov and Dobrovolskij 2013), as well as, to some extent, cercariae of *Trichobilharzia* sp., which are known to attach to the walls of experimental vessels (Stanicka et al. 2021a). However, cercariae of *Diplostomum* sp. and most of *Trichobilharzia* sp. primarily remain suspended in the water column (Haas et al. 2008; Stanicka et al. 2021a). Accordingly, large snails consumed *Notocotylus* sp. adolescariae most efficiently and *Diplostomum* sp. cercariae least efficiently. Similar conclusions were drawn by Selbach et al. (2019), who found that *P. acuta* consumed only bottom-dwelling cercarial species, but not free-swimming ones.

On the other hand, small *P. acuta* individuals consumed more cercariae than adolescariae. A possible explanation for this may be that smaller snails need to surface more frequently to obtain air. This behaviour likely increases their chance of encountering cercariae suspended in the water column (Stanicka et al. 2021a), while simultaneously reducing their exposure to larvae settled on the substratum.

### The effect of metacercarial burden on snail foraging

An increase in prey consumption was observed in snails infected with xiphidiometacercariae. This pattern may reflect the elevated energy demands commonly associated with parasitic infections (Kuris and Warren 1980), which often trigger more intensive foraging behaviour in hosts (Shinagawa et al. 2001; Díaz-Morales et al. 2023). Notably, parasitised *P. acuta* were more effective at consuming adolescariae, the encysted and inactive form of the parasite.

Although metacercariae are generally considered to be of low pathogenicity (Ballabeni 1994; Shirakashi et al. 2020), some studies report their negative effects on host condition and survival. These impacts depend on factors such as infection intensity and parasite localisation (Majoros 1999; Ogawa et al. 2004; Bates et al. 2010; Varas et al. 2022) and may include altered behaviour that increases susceptibility to predation by the final host (Seppälä et al. 2004). For example, Martin and Conn (1990) demonstrated that echinostome metacercariae can impair kidney function in frog hosts.

Furthermore, the process of active transmission – i.e. the simultaneous penetration of the host’s body surface by numerous cercariae – can be harmful to second intermediate hosts (Kuris and Warren 1980; Majoros 1999; Bates et al. 2010). The subsequent migration of larvae through tissues and organs before encystment also poses risks (Islam 1986; Majoros 1999).

Therefore, it is reasonable to assume that even metacercarial infections – especially as intense as those observed in this study – can influence host fitness and mobility, ultimately affecting their foraging efficiency across different prey types.

### The effect of the oligochaete presence on snail foraging

The presence of *C. limnaei limnaei* led to increased consumption of *Diplostomum* sp. cercariae and decreased consumption of *Notocotylus* sp. adolescariae. This outcome is most likely attributable to the combined feeding activity of both *P. acuta* and *C. limnaei limnaei*. Cercariae have previously been observed in the digestive tract of *C. limnaei limnaei* (Khalil 1961; Fashuyi and Williams 1977; Fried et al. 2008), indicating that the oligochaete can directly ingest these larvae.

Although *C. limnaei limnaei* is capable of temporarily leaving its snail host (Sankurathri and Holmes 1976), no oligochaetes were found on membrane filters during larval counting. This suggests that the worms remained within the snails and only protruded from the mantle cavity, as previously observed (Sankurathri and Holmes 1976). Such behaviour is likely associated with the consumption of larvae suspended in the water column, such as *Diplostomum* sp. cercariae.

In addition to direct feeding, experimental studies have shown that *C. limnaei limnaei* can influence the behaviour of its host, including foraging activity (Stoll et al. 2013). At high infestation levels, *P. acuta* individuals tend to spend less time foraging and more time resting (Stoll et al. 2013). This behavioural change may account for the reduced consumption of settled *Notocotylus* sp. adolescariae, which are not targeted by the oligochaete.

### *Physa acuta* as a host for digenean larvae

The limited role of non-native and invasive species in parasite transmission within introduced ranges is frequently emphasised (Ebbs et al. 2018). However, in the present study, *P. acuta* commonly functioned as a second intermediate host for digeneans. The detection of digenean metacercariae in *P. acuta* raises several key research questions: (I) Can non-native parasites be co-introduced with this invasive snail and subsequently infect native host species (*parasite spillover*)? (II) Does *P. acuta* serve as an alternative host for native or previously established parasites, potentially increasing their transmission (*parasite spillback*)?

Without molecular data, the precise taxonomic identification of Digenea, especially those isolated from intermediate hosts, remains difficult due to substantial intra-generic morphological similarity and high species-level variability (Dvorak et al. 2002; Kudlai et al. 2021; Pantoja et al. 2021). Nevertheless, based on morphological characteristics of the metacercariae observed in this study and comparative data from the literature (Graczyk and Shiff 1993; Faltýnková et al. 2007; Cichy and Żbikowska 2016; Stanicka et al. 2020; Bespalaya et al. 2022; Kanarek et al. 2023), the digenean species associated with *P. acuta* in its non-native range appear to belong to taxa that are common and widely distributed in Europe. Therefore, based on the present study, *P. acuta* can be involved in *parasite spillback* in Europe. However, it should also be considered that for some digenean species, *P. acuta* may act as a dead-end host.

## Conclusion

Our study demonstrates that the non-native snail *P. acuta* can interact with free-living stages of digenean parasites, diluting both cercariae and adolescariae. These findings highlight its dual ecological role – not only as a potential host for metacercariae but also as a consumer that may reduce the abundance of infective larval stages in the environment. In addition, the commensal oligochaete *C. limnaei limnaei* associated with *P. acuta* may further contribute to the dilution of cercariae through its own feeding activity. Given the widespread distribution, ecological plasticity, and often high densities of *P. acuta*, such interactions could modify parasite transmission dynamics and energy flow within freshwater ecosystems. This study provides insight into how non-native species and their symbionts can jointly shape host–parasite–commensal networks, with potential consequences at the community and ecosystem levels. Future research linking individual-level feeding behaviour with ecosystem-scale outcomes would help better understand these complex trophic relationships.

## Supporting information

10.1017/S0031182025101169.sm001 Stanicka et al. supplementary material Stanicka et al. supplementary material

## Data Availability

The data that support the findings of this study are available from the corresponding author, AS, upon reasonable request.

## Supplementary material

The supplementary material for this article can be found at https://doi.org/10.1017/S0031182025101169.

## References

[ref1] Al-Jubury A, Kania P, Bygum A and Buchmann K (2020) Temperature and light effects on *Trichobilharzia szidati* cercariae with implications for a risk analysis. *Acta Veterinaria Scandinavica* 62, 1–9. 32933558 10.1186/s13028-020-00553-z PMC7493345

[ref2] Antychowicz J, Bernat A, Kramer I, Glowacka H and Pekala A (2016) Pasożyty europejskich wolno żyjących ryb śródlądowych, ze szczególnym uwzględnieniem występujących w polskich jeziorach i rzekach. *Zycie Weterynaryjne* 91, 549–560.

[ref3] Ballabeni P (1994) Experimental differences in mortality patterns between European minnows, *Phoxinus phoxinus*, infected with sympatric or allopatric trematodes, *Diplostomum phoxini*. *Journal of Fish Biology* 45, 257–267. 10.1111/j.1095-8649.1994.tb01305.x.

[ref4] Bashê SK (2023) Ecology, morphology and molecular confirmation of **Chaetogaster limnaei** (Annelida: Naididae) retrieved from freshwater snail *Physa acuta* from Greater Zab River, Iraq. *Tikrit Journal of Pure Science* 28, 21–26.

[ref5] Bates AE, Poulin R and Lamare MD (2010) Spatial variation in parasite-induced mortality in an amphipod: Shore height versus exposure history. *Oecologia* 163, 651–659. 10.1007/s00442-010-1593-5. 20217140

[ref6] Bespalaya YV, Kondakov AV, Travina OV, Khrebtova IS, Kropotin AV, Aksenova OV, Gofarov MY, Lyubas AA, Tomilova AA and Vikhrev IV (2022) First record of metacercariae trematodes *Opisthioglyphe ranae* (Digenea: Telorchiidae) and *Echinostoma bolschewense* (Digenea: Echinostomatidae) in *Dreissena polymorpha* (Bivalvia: Dreissenidae) from the Don and Volga river basins, Russia. *Ecologica Montenegrina* 54, 57–76.

[ref7] Born-Torrijos A, Paterson RA, van Beest GS, Vyhlídalová T, Henriksen EH, Knudsen R, Kristoffersen R, Amundsen P-A and Soldánová M (2021) Cercarial behaviour alters the consumer functional response of three-spined sticklebacks. *Journal of Animal Ecology* 90, 978–988. 33481253 10.1111/1365-2656.13427

[ref8] Braun L, Grimes JE and Templeton MR (2018) The effectiveness of water treatment processes against schistosome cercariae: A systematic review. *PLoS Neglected Tropical Diseases* 12, e0006364. 29608589 10.1371/journal.pntd.0006364 PMC5903662

[ref9] Cichy A and Żbikowska E (2016) *Atlas of Digenea Developmental Stages: The Morphological Characteristics and Spread within the Populations of Freshwater Snails from the Brodnickie Lakeland, Poland*. Toruń: Wydawnictwo Naukowe Uniwersytetu Mikołaja Kopernika.

[ref10] Cieplok A and Spyra A (2020) The roles of spatial and environmental variables in the appearance of a globally invasive *Physa acuta* in water bodies created due to human activity. *Science of the Total Environment* 744, 140928. 10.1016/j.scitotenv.2020.140928. 32698048

[ref11] Cribb TH (1991) Notocotylidae (Digenea) from the Australian water rat *Hydromys chrysogaster* Geoffroy, 1804 (Muridae). *Systematic Parasitology* 18, 227–237. 10.1007/BF00009362.

[ref12] Díaz-Morales DM, Bommarito C, Knol J, Grabner DS, Noè S, Rilov G, Wahl M, Guy-Haim T and Sures B (2023) Parasitism enhances gastropod feeding on invasive and native algae while altering essential energy reserves for organismal homeostasis upon warming. *Science of the Total Environment* 863, 160727. 10.1016/j.scitotenv.2022.160727. 36502976

[ref13] Díaz-Morales DM, Sures B, Jolma ER and Thieltges DW (2025) Invasion biology in the context of aquatic host–parasite interactions. In Smit NJ and Sures B (eds), *Aquatic Parasitology: ecological and Environmental Concepts and Implications of Marine and Freshwater Parasites*. Cham, Switzerland: Springer Nature 471-491.

[ref14] Dunn AM, Torchin ME, Hatcher MJ, Kotanen PM, Blumenthal DM, Byers JE, Coon CA, Frankel VM, Holt RD and Hufbauer RA (2012) Indirect effects of parasites in invasions. *Functional Ecology* 26, 1262–1274.

[ref15] Dvorak J, Vanacova S, Hampl V, Flegr J and Horák P (2002) Comparison of European *Trichobilharzia* species based on ITS1 and ITS2 sequences. *Parasitology* 124, 307. 11922432 10.1017/s0031182001001238

[ref16] Ebbs ET, Loker ES and Brant SV (2018) Phylogeography and genetics of the globally invasive snail *Physa acuta* Draparnaud 1805, and its potential to serve as an intermediate host to larval digenetic trematodes. *BMC Evolutionary Biology* 18, 103. 10.1186/s12862-018-1208-z. 29969987 PMC6029401

[ref17] Faltýnková A and Haas W (2006) Larval trematodes in freshwater molluscs from the Elbe to Danube rivers (Southeast Germany): Before and today. *Parasitology Research* 99, 572–582. 10.1007/s00436-006-0197-9. 16670883

[ref18] Faltýnková A, Našincová V and Kablásková L (2007) Larval trematodes (Digenea) of the great pond snail, *Lymnaea stagnalis* (L.),(Gastropoda, Pulmonata) in Central Europe: A survey of species and key to their identification. *Parasite* 14, 39–51. 17432056 10.1051/parasite/2007141039

[ref19] Faltýnková A, Našincová V and Kablásková L (2008) Larval trematodes (Digenea) of planorbid snails (Gastropoda: Pulmonata) in Central Europe: A survey of species and key to their identification. *Systematic Parasitology* 69, 155–178. 18210216 10.1007/s11230-007-9127-1

[ref20] Fashuyi SA and Williams MO (1977) The role of *Chaetogaster limnaei* in the dynamics of trematode transmission in natural populations of freshwater snails. *Zeitschrift Für Parasitenkunde* 54, 55–60. 10.1007/BF00380636. 602368

[ref21] Flores VR, Hernández-Orts JS and Viozzi GP (2023) A new species of *Notocotylus* (Digenea: Notocotylidae) from the black-necked swan *Cygnus melancorhyphus* (Molina) of Argentina. *Veterinary Parasitology: Regional Studies and Reports* 45, 100925. 37783528 10.1016/j.vprsr.2023.100925

[ref22] Fried B, Peoples RC, Saxton TM and Huffman JE (2008) The association of *Zygocotyle lunata* and *Echinostoma trivolvis* with *Chaetogaster limnaei*, an ectosymbiont of *Helisoma trivolvis*. *Journal of Parasitology* 94, 553–554. 18564763 10.1645/GE-1388.1

[ref23] Galaktionov KV and Dobrovolskij A (2013) *The Biology and Evolution of Trematodes: an Essay on the Biology, Morphology, Life Cycles, Transmissions, and Evolution of Digenetic Trematodes*. Dordrecht: Springer Science & Business Media.

[ref24] Gonchar A, Jouet D, Skírnisson K, Krupenko D and Galaktionov KV (2019) Transatlantic discovery of *Notocotylus atlanticus* (Digenea: Notocotylidae) based on life cycle data. *Parasitology Research* 118, 1445–1456. 10.1007/s00436-019-06297-8. 30919063

[ref25] Graczyk TK and Shiff CJ (1993) Experimental infection of domestic ducks and rodents by *Notocotylus attenuatus* (Trematoda: Notocotylidae). *Journal of Wildlife Diseases* 29, 434–439. 8355345 10.7589/0090-3558-29.3.434

[ref26] Haas W, Beran B and Loy C (2008) Selection of the host’s habitat by cercariae: From laboratory experiments to the field. *Journal of Parasitology* 94, 1233–1238. 10.1645/GE-1192.1. 18576700

[ref27] Hobart BK, Moss WE, McDevitt-Galles T, Stewart Merrill TE and Johnson PT (2021) It’s a worm-eat-worm world: Consumption of parasite free-living stages protects hosts and benefits predators. *Journal of Animal Ecology* 91, 35–45. 34543447 10.1111/1365-2656.13591

[ref28] Hopper JV, Poulin R and Thieltges DW (2008) Buffering role of the intertidal anemone *Anthopleura aureoradiata* in cercarial transmission from snails to crabs. *Journal of Experimental Marine Biology and Ecology* 367, 153–156.

[ref29] Islam KS (1986) The morphology and life-cycle of *Trichobilharzia arcuata* n. sp.(Schistosomatidae: Bilharziellinae) a nasal schistosome of water whistle ducks (*Dendrocygna arcuata*) in Australia. *Systematic Parasitology* 8, 117–128.

[ref30] Jeschke JM, Bacher S, Blackburn TM, Dick JTA, Essl F, Evans T, Gaertner M, Hulme PE, Kühn I, Mrugała A, Pergl J, Pyšek P, Rabitsch W, Ricciardi A, Richardson DM, Sendek A, Vilà M, Winter M and Kumschick S (2014) Defining the impact of non-native species. *Conservation Biology* 28, 1188–1194. 10.1111/cobi.12299. 24779412 PMC4282110

[ref31] Johnson PTJ, Dobson A, Lafferty KD, Marcogliese DJ, Memmott J, Orlofske SA, Poulin R and Thieltges DW (2010) When parasites become prey: Ecological and epidemiological significance of eating parasites. *Trends in Ecology and Evolution* 25, 362–371. 10.1016/j.tree.2010.01.005. 20185202

[ref32] Kanarek G, Gabrysiak J, Pyrka E, Jeżewski W, Stanicka A, Cichy A, Żbikowska E, Zaleśny G and Hildebrand J (2023) Hyperparasitism among larval stages of Digenea in snail hosts: Sophisticated life strategy or pure randomness? The scenario of Cotylurus sp. *Zoological Journal of the Linnean Society* 200, 865–875.

[ref33] Karvonen A, Paukku S, Valtonen ET and Hudson PJ (2003) Transmission, infectivity and survival of *Diplostomum spathaceum* cercariae. *Parasitology* 127, 217–224. 10.1017/S0031182003003561. 12964824

[ref34] Karvonen A and Seppälä O (2008) Eye fluke infection and lens size reduction in fish: A quantitative analysis. *Diseases of Aquatic Organisms* 80, 21–26. 18714680 10.3354/dao01918

[ref35] Khalil LF (1961) On the capture and destruction of miracidia by *Chaetogaster limnaei* (Oligochaeta). *Journal of Helminthology* 35, 269–274. 14455522 10.1017/s0022149x00004648

[ref36] Koprivnikar J, Thieltges D and Johnson P (2023) Consumption of trematode parasite infectious stages: From conceptual synthesis to future research agenda. *Journal of Helminthology* 97, e33. 10.1017/S0022149X23000111. 36971341 PMC12594072

[ref37] Kudlai O, Pantoja C, O’Dwyer K, Jouet D, Skírnisson K and Faltýnková A (2021) Diversity of *Plagiorchis* (Trematoda: Digenea) in high latitudes: Species composition and snail host spectrum revealed by integrative taxonomy. *Journal of Zoological Systematics and Evolutionary Research* 59, 937–962.

[ref38] Kuris AM and Warren J (1980) Echinostome cercarial penetration and metacercarial encystment as mortality factors for a second intermediate host, *Biomphalaria glabrata*. *The Journal of Parasitology* 66, 630–635. 7420245

[ref39] Majoros G (1999) Mortality of fish fry as a result of specific and aspecific cercarial invasion under experimental conditions. *Acta Veterinaria Hungarica* 47, 433–450. 10641334 10.1556/AVet.47.1999.4.4

[ref40] Marszewska A, Cichy A, Heese T and Żbikowska E (2016) The real threat of swimmers’ itch in anthropogenic recreational water body of the Polish Lowland. *Parasitology Research* 115, 3049–3056. 27083184 10.1007/s00436-016-5060-z PMC4958134

[ref41] Martin TR and Conn DB (1990) The pathogenicity, localization, and cyst structure of echinostomatid metacercariae (Trematoda) infecting the kidneys of the frogs *Rana clamitans* and Rana pipiens. *The Journal of Parasitology* 76, 414–419. 2352071

[ref42] Mas-Coma S, Valero MA and Bargues MD (2015) Fasciola and Fasciolopsis. In Xiao L, Ryan U and Feng Y (eds), *Biology of Foodborne Parasites*. Boca Raton, FL: CRC Press, pp. 371–404.

[ref43] McKee KM, Koprivnikar J, Johnson PT and Arts MT (2020) Parasite infectious stages provide essential fatty acids and lipid-rich resources to freshwater consumers. *Oecologia* 192, 477–488. 31834514 10.1007/s00442-019-04572-0

[ref44] Morley NJ, Crane M and Lewis JW (2002) Toxicity of cadmium and zinc to encystment of *Notocotylus attenuatus* (Trematoda: Notocotylidae) cercariae. *Ecotoxicology & Environmental Safety* 53, 129–133. 12481868 10.1006/eesa.2002.2198

[ref45] Ogawa K, Nakatsugawa T and Yasuzaki M (2004) Heavy metacercarial infections of cyprinid fishes in Uji River. *Fisheries Science* 70, 132–140.

[ref46] Okeke O and Ubachukwu P (2017) Cooccurrence of *Schistosoma haematobium*, other trematode parasites, an annelid (*Chaetogaster limnaei limnaei*), and a nematode parasite (*Daubaylia potomaca*) in *Bulinus globosus*. *Turkish Journal of Zoology* 41, 196–202.

[ref47] Pantoja C, Faltýnková A, O’Dwyer K, Jouet D, Skírnisson K and Kudlai O (2021) Diversity of echinostomes (Digenea: Echinostomatidae) in their snail hosts at high latitudes. *Parasite* 28, 59. 34319230 10.1051/parasite/2021054 PMC8336728

[ref48] Piechocki A (1979) *Mieczaki (Mollusca). Slimaki (Gastropoda)*. Warszawa: Panstwowe Wydawnictwo Naukowe.

[ref49] Piechocki A and Wawrzyniak-Wydrowska B (2016) *Guide to Freshwater and Marine Mollusca of Poland*. Poznań: Bogucki Wydawnictwo Naukowe.

[ref50] Rosen R, Staat S, Andrews M, Budhathoki Y, Jackson H, Kwisera B and Mecham J (2025) Additional predators (Nonhosts) and a new amphibian host of the digenetic trematode cercaria of *Proterometra macrostoma* in laboratory experiments. *Comparative Parasitology* 92, 56–61.

[ref51] Sankurathri CS and Holmes JC (1976) Effects of thermal effluents on parasites and commensals of *Physa gyrina* Say (Mollusca: Gastropoda) and their interactions at Lake Wabamun, Alberta. *Canadian Journal of Zoology* 54, 1742–1753. 10.1139/z76-202.

[ref52] Schatz AM and Park AW (2023) Patterns of host–parasite coinvasion promote enemy release and specialist parasite spillover. *Journal of Animal Ecology* 92, 1029–1041. 36934311 10.1111/1365-2656.13910

[ref53] Selbach C, Rosenkranz M and Poulin R (2019) Cercarial behavior determines risk of predation. *Journal of Parasitology* 105, 330–333. 31021737

[ref54] Seppälä O, Karvonen A and Tellervo Valtonen E (2004) Parasite-induced change in host behaviour and susceptibility to predation in an eye fluke–fish interaction. *Animal Behaviour* 68, 257–263. 10.1016/j.anbehav.2003.10.021.

[ref55] Shinagawa K, Urabe M and Nagoshi M (2001) Effects of trematode infection on metabolism and activity in a freshwater snail, *Semisulcospira libertina*. *Diseases of Aquatic Organisms* 45, 141–144. 11463101 10.3354/dao045141

[ref56] Shirakashi S, Waki T and Ogawa K (2020) Bucephalid metacercarial infection in wild larval and juvenile ayu *Plecoglossus altivelis*. *Fish Pathology* 54, 93–100.

[ref57] Simon-Vicente F, Mas-Coma S, Lopez-Roman R, Tenora F and Gallego J (1985) Biology of *Notocotylus neyrai* Gonzalez Castro, 1945 (Trematoda). *Folia Parasitologica* 32, 101–111.

[ref58] Soldánová M, Faltýnková A, Scholz T and Kostadinova A (2011) Parasites in a man-made landscape: Contrasting patterns of trematode flow in a fishpond area in Central Europe. *Parasitology* 138, 789–807. 10.1017/S0031182011000291. 24650935

[ref59] Soldánová M, Selbach C, Kalbe M, Kostadinova A and Sures B (2013) Swimmer’s itch: Etiology, impact, and risk factors in Europe. *Trends in Parasitology* 29, 65–74. 10.1016/j.pt.2012.12.002. 23305618

[ref60] Spyra A, Cieplok A, Strzelec M and Babczyńska A (2019) Freshwater alien species *Physella acuta* (Draparnaud, 1805) – A possible model for bioaccumulation of heavy metals. *Ecotoxicology & Environmental Safety* 185, 109703. 31561074 10.1016/j.ecoenv.2019.109703

[ref61] Stanicka A, Cichy A, Bulantová J, Labecka AM, Ćmiel AM, Templin J, Horák P and Żbikowska E (2022) Thinking “outside the box”: The effect of nontarget snails in the aquatic community on mollusc-borne diseases. *Science of the Total Environment* 845, 157264. 35820526 10.1016/j.scitotenv.2022.157264

[ref62] Stanicka A, Dlouhy Z, Cichy A, Żbikowska E and Jermacz Ł (2025) In the face of fear: The success of encounters between digenean cercariae and an intermediate target host under predation pressure. *International Journal for Parasitology* 10, 547–555. 10.1016/j.ijpara.2025.04.012. 40252799

[ref63] Stanicka A, Migdalski Ł, Szopieray K, Cichy A, Jermacz Ł, Lombardo P and Żbikowska E (2021a) Invaders as diluents of the cercarial dermatitis etiological agent. *Pathogens* 10, 740. 34208370 10.3390/pathogens10060740 PMC8231267

[ref64] Stanicka A, Migdalski Ł, Zając KS, Cichy A, Lachowska-Cierlik D and Żbikowska E (2021b) The genus *Bilharziella* vs. other bird schistosomes in snail hosts from one of the major recreational lakes in Poland. *Knowledge & Management of Aquatic Ecosystems* 422, 12.

[ref65] Stanicka A, Soldánová M, Migdalski Ł, Szopieray K, Lesiak K, Cichy A, Żbikowska E and Jermacz Ł (2023) New species, new story: The impact of invasive non-host predators on host-trematode interactions. *Freshwater Biology* 68, 1–12. 10.1111/fwb.14155.

[ref66] Stanicka A, Zając KS, Lachowska-Cierlik D, Cichy A, Żbikowski J and Żbikowska E (2020) Potamopyrgus antipodarum (Gray, 1843) in Polish waters – its 25 mitochondrial haplotype and role as intermediate host for trematodes. *Knowledge & Management of Aquatic Ecosystems* 421, 48.

[ref67] Stoll S, Früh D, Westerwald B, Hormel N and Haase P (2013) Density-dependent relationship between *Chaetogaster limnaei limnaei* (Oligochaeta) and the freshwater snail *Physa acuta* (Pulmonata). *Freshwater Science* 32, 642–649. 10.1899/12-072.1.

[ref68] Varas O, Pulgar J, Duarte C, García-Herrera C, Abarca-Ortega A, Grenier C, Rodríguez-Navarro AB, Zapata J, Lagos NA, García-Huidobro MR and Aldana M (2022) Parasitism by metacercariae modulates the morphological, organic and mechanical responses of the shell of an intertidal bivalve to environmental drivers. *Science of the Total Environment* 830, 154747. 10.1016/j.scitotenv.2022.154747. 35337870

[ref69] Wauters LA, Lurz PW, Santicchia F, Romeo C, Ferrari N, Martinoli A and Gurnell J (2023) Interactions between native and invasive species: A systematic review of the red squirrel-gray squirrel paradigm. *Frontiers in Ecology and Evolution* 11, 1083008.

[ref70] Welsh JE, Hempel A, Markovic M, Van Der Meer J and Thieltges DW (2019) Consumer and host body size effects on the removal of trematode cercariae by ambient communities. *Parasitology* 146, 342–347. 30318030 10.1017/S0031182018001488

