# Supplementary material for: Who eats whom? Interactions between the non-native snail Physa acuta, local digeneans, and a commensal oligochaete
Source: Parasitology. 2025 Nov 10;153(1):13–21. doi: 10.1017/S0031182025101169 (PMC13215724; doi:10.1017/S0031182025101169)
Supplement: Stanicka et al. supplementary material [file S0031182025101169sup001.docx]

**Who eats whom? Interactions between the non-native snail *Physa acuta*, local digeneans, and a commensal oligochaete**

Anna Stanicka^1^, Jarosław Kobak^1^, Zuzanna Kowaleska^1^, Monika Lewalska^1^, Wiktoria Pacek^1^, Arkadiusz Grzeczka^2^, Szymon Graczyk^2^, Anna Cichy^1^ and Elżbieta Żbikowska^1^

^1^Department of Invertebrate Zoology and Parasitology, Nicolaus Copernicus University in Toruń, Toruń, Poland; ^2^Institute of Veterinary Medicine, Nicolaus Copernicus University in Toruń, Toruń, Poland

**Corresponding author:** Anna Stanicka, Email: anna.marszewska@umk.pl

Table SM1. Post-hoc comparisons for significant interactions of digenean larva taxon with (A) snail size group, (B) xyphidiometacercariae abundance and (C) *Chaetogaster limnaei limnaei* abundance in the Generalised Linear Model to test consumption of digenean larvae by snails in various treatments (see Table 1 for the main model results). For significant interactions involving continuous covariates (B-C), we first checked the significance of regression slopes for each digenean larva species and then compared significant slopes with each other.

| Digenean larva taxon | A  Digenean larva taxon*snail size | |  | B  Digenean larva taxon*Xyphidiometacercariae | |  | C  Digenean larva taxon**Ch. limnaei limnaei* | |
| --- | --- | --- | --- | --- | --- | --- | --- | --- |
|  | Small vs large | |  | Significance of slopes | | | | |
|  |  |  |  | Wald χ^2^_1_ | P |  | Wald χ^2^_1_ | P |
| *Diplostomum* sp. (D) | 0.021 | |  | 5.57 | 0.018 |  | 20.56 | <0.001 |
| *Trichobilharzia* sp. (T) | <0.001 | |  | 5.80 | 0.016 |  | 1.15 | 0.284 |
| *Notocotylus* sp. (N) | <0.001 | |  | 133.60 | <0.001 |  | 36.28 | <0.001 |
|  |  |  |  |  | | | | |
|  | Small | Large |  | Differences between significant slopes | | | | |
|  |  |  |  | Wald χ^2^_1_ | P |  | Wald χ^2^_1_ | P |
| D *vs* T | 0.128 | <0.001 |  | 13.50 | <0.001 |  |  |  |
| D *vs* N | <0.001 | <0.001 |  | 128.08 | <0.001 |  | 62.17 | <0.001 |
| T *vs* N | 0.002 | <0.001 |  | 90.91 | <0.001 |  |  |  |


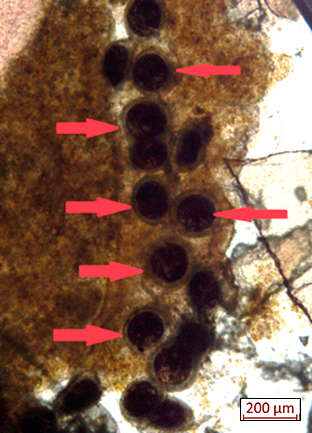


Figure SM1. Adolescariae of *Notocotylus* sp. (indicated by arrows) in the digestive system of experimental *Physa acuta.*


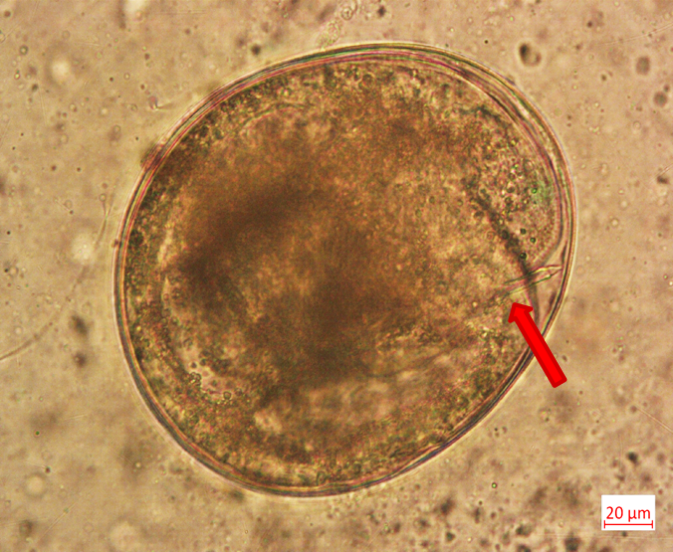


Figure SM2. Xyphidiometacercaria with a visible stylet with a characteristic base (arrow).
